# Manifesto on small airway involvement and management in asthma and chronic obstructive pulmonary disease: an Interasma (Global Asthma Association - GAA) and World Allergy Organization (WAO) document endorsed by Allergic Rhinitis and its Impact on Asthma (ARIA) and Global Allergy and Asthma European Network (GA^2^LEN)

**DOI:** 10.1186/s40733-016-0027-5

**Published:** 2016-10-28

**Authors:** F. Braido, N. Scichilone, F. Lavorini, O. S. Usmani, L. Dubuske, L. P. Boulet, R. Mosges, C. Nunes, M. Sánchez-Borges, I. J. Ansotegui, M. Ebisawa, F. Levi-Schaffer, L. J Rosenwasser, J. Bousquet, T. Zuberbier, G. Walter Canonica, for the Interasma Executive Board, A. Cruz, A. Yanez, A. Yorgancioglu, D. Deleanu, G. Rodrigo, J. Berstein, K. Ohta, P. Vichyanond, S. Gonzales Diaz, S. Nakajima, T. Slavyanskaya, A. Fink-Wargner, C. Baez Loyola, D. Ryan, G. Passalacqua, J. Celedon, J. C. Invancevich, K. Dobashi, M. Zernotti, WAO Board of Directors, R. Pawankar, M. Zernotti, P. Vichyanond, M. Akdis, S. Benjaponpitak, S. Bonini, W. Burks, L. Caraballo, Z. Awad El-Sayed, S. Fineman, P. Greenberger, E. Hossny, J. A. Ortega-Martell, H. Saito, M. Tang, L. Zhang, ARIA, J. Bousquet, GA²LEN, T. Zuberbier

**Affiliations:** 1 Allergy and Respiratory Diseases Department DIMI, University of Genoa, IRCCS AOU San Martino-IST, Genoa, Italy; 2 Dipartimento Biomedico di Medicina Interna e Specialistica, University of Palermo, Palermo, Italy; 3 Department of Experimental and Clinical Medicine, University of Florence, Florence, Italy; 4 Airway Disease Section, National Heart and Lung Institute, Imperial College London, Royal Brompton Hospital, London, UK; 5 Immunology Research Institute of New England, Harvard, USA; 6 Centre de recherche de l’Institut universitaire de cardiologie et de pneumologie de Québec, Québec, Canada; 7 Institute of Medical Statistics, Informatics and Epidemiology, University Hospital of Cologne, Cologne, Germany; 8 Centro de ImmunoAlergologia de Algarve, Porto, Portugal; 9 Centro Medico Docente La Trinidad, Caracas, Venezuela; 10 Clinica El Avila, Caracas, Venezuela; 11 Department of Allergy and Immunology, Hospital Quirón Bizkaia, Carretera Leioa-Inbe, Erandio, Bilbao, Spain; 12 Department of Allergy, Clinical Research Center for Allergy & Rheumatology, Sagamihara National Hospital, Sagamihara, Kanagawa Japan; 13 Department of Pharmacology and Experimental Therapeutics, Institute for Drug Research, School of Pharmacy, Faculty of Medicine, The Hebrew University of Jerusalem, Jerusalem, Israel; 14 University of Missouri - Kansas City, School of Medicine, Kansas City, Missouri USA; 15 Service des Maladies Respiratoires, Hopital Arnaud de Villeneuve, Montpellier, France; 16 Department of Dermatology and Allergy, Charité-Universitätsmedizin Berlin, Berlin, Germany

## Abstract

Evidence that enables us to identify, assess, and access the small airways in asthma and chronic obstructive pulmonary disease (COPD) has led INTERASMA (Global Asthma Association) and WAO to take a position on the role of the small airways in these diseases.

Starting from an extensive literature review, both organizations developed, discussed, and approved the manifesto, which was subsequently approved and endorsed by the chairs of ARIA and GA^2^LEN. The manifesto describes the evidence gathered to date and defines and proposes issues on small airway involvement and management in asthma and COPD with the aim of challenging assumptions, fostering commitment, and bringing about change.

The small airways (defined as those with an internal diameter <2 mm) are involved in the pathogenesis of asthma and COPD and are the major determinant of airflow obstruction in these diseases. Various tests are available for the assessment of the small airways, and their results must be integrated to confirm a diagnosis of small airway dysfunction.

In asthma and COPD, the small airways play a key role in attempts to achieve disease control and better outcomes. Small-particle inhaled formulations (defined as those that, owing to their size [usually <2 μm], ensure more extensive deposition in the lung periphery than large molecules) have proved beneficial in patients with asthma and COPD, especially those in whom small airway involvement is predominant.

Functional and biological tools capable of accurately assessing the lung periphery and more intensive use of currently available tools are necessary. In patients with suspected COPD or asthma, small airway involvement must be assessed using currently available tools. In patients with subotpimal disease control and/or functional or biological signs of disease activity, the role of small airway involvement should be assessed and treatment tailored. Therefore, the choice between large- and small-particle inhaled formulations must reflect the physician’s considerations of disease features, phenotype, and response to previous therapy.

This article is being co-published in *Asthma Research and Practice* and the *World Allergy Organization Journal*.

## Background

A manifesto, from the Latin “manifestum” (meaning clear or evident), is a declaration of the beliefs, opinions, motives, and intentions of the issuer, be it an individual or a group. It is normally based on previously published opinion or public consensus and attempts to promote new ideas with prescriptive notions. In the context of health care, a manifesto describes confirmed evidence, actions required, and investigations necessary and is issued by a group of experts or a scientific organization on a specific topic. By leading people to evaluate the gap between these principles and their current reality, the manifesto challenges assumptions, fosters commitment, and provokes change.

The involvement of the small airways of the lung (“silent zone”) in the pathogenesis of asthma and chronic obstructive pulmonary disease (COPD) is currently the object of much research and debate, and the ability to identify, assess, and access the small airways enables us to take a position on this timely issue in the light of available evidence.

We performed a PubMed search using the MeSH terms “small airway” and “small particle” within the last three years (January 2013 to January 2016). We also used the key words “large”, “fine”, “small”, “respirable”, “extra-fine”, “extra-small particle”, together with the MeSh terms “asthma/COPD”, which were limited to clinical trials without time limits. All selected papers were initially evaluated by a panel of experts to assess their eligibility in contributing to the statements of the manifesto. In February 2016, the draft of the manifesto was circulated among the Board of Officers of Interasma (Global Asthma Association) and the WAO Board of Directors, who appraised, discussed, and approved the final version. The final version of the Manifesto was approved and endorsed by the chairs of ARIA^§^ and GA^2^LEN^.

### We define

Small airways as those with an internal diameter <2 μm [1].

Drug particles smaller than 5 μm are called “large particles”; “small particles” are defined as particles whose size (usually <2 μm) enables them to be depositied in the lung periphery in greater amounts than large molecules [2–6].

### We know

That while the small airways contribute little to total airway resistance in healthy lungs, in asthma and in COPD they are the major determinant of airflow obstruction [7, 8].

It has been estimated that 75 % of the small airways must be obstructed before changes can be detected using routine pulmonary function tests (e.g. forced expiratory volume in 1 second [FEV_1_]). [9].

Various tests are available for the assessment of small airways. The detection of an abnormal forced expiratory flow (FEF_25–75_) should be supported by other pulmonary function tests (impulse oscillometry, whole-body plethysmography, exhaled-breath nitric oxide, and single-breath and multiple-breath nitrogen washout) to confirm a diagnosis of small airway dysfunction [4, 10–12].

#### Asthma

The small airways play a role in the pathobiology of asthma and, although they are involved in half of all cases of asthma, they can have a distinct role in specific disease phenotypes [13–17].

Inflammatory changes involving the small airways influence the severity of asthma [18–23].

Functional alterations of the small airways are also associated with the severity of asthma [24–32].

The role of the small airways in asthma is increasingly recognized as a potential target in optimal control of the disease [33–36].

The development of small-particle inhaled formulations enables the drug to reach both the large and the small airways [37–39].

Small-particle aerosols have been shown to be as efficacious as large-particle aerosols in randomized controlled trials; however, real-life studies have shown that smaller particles are more efficacious and improve asthma control and quality of life compared with large particles; in addition, they do so with a marked reduction in the daily dose of inhaled corticosteroids (ICS) [37, 40–75].

#### COPD

COPD is characterized by a mixture of small airway impairment (obstructive bronchitis/bronchiolitis) and parenchymal lung tissue destruction (emphysema). The progression of COPD is strongly associated with small airway wall thickening as a result of lung repair or remodelling [76–79].

Small airway disease in COPD can be identified by pulmonary function testing, multiple nitrogen breath washout testing, indirectly through high-resolution chest computed tomography (CT) imaging or inert-gas MRI imaging, or directly using microCT of resected lung tissue [80, 81].

In COPD, air trapping and functional small airway impairment are associated with inflammation of the peripheral airways [82–84].

The effects of significantly low-dose small-particle ICS on exacerbation rates are similar to those of larger-particle ICS at higher doses. Initiation of small-particle ICS is associated with a greater likelihood of stable treatment [85–87].

The combination of small-particle ICS and long-acting beta-agonists has been effective in reducing air trapping and improving health-related quality of life and dyspnea in COPD patients with lung hyperinflation [88–94].

### We state

That small airways play a key role in the pathogenesis of asthma and COPD.

In asthma and in COPD, the small airways can be targeted to ensure disease control and better outcomes.

Small-particle inhaled formulations are beneficial in patients with asthma and COPD, especially those with predominant small airway involvement.

### We advocate

The need for functional and biological tools that can accurately assess the lung periphery and a more intensive use of currently available tools for improved patient phenotyping.

The lack of consensus on the terminology used to describe small drug particles necessitates a clear nomenclature to ensure that uniform terminology is used by professionals.

Research should aim to identify a specific “small airway phenotype” so that uncontrolled disease can be treated appropriately.

### We propose

That in patients with suspected COPD or asthma, small airway involvement should be taken into consideration and explored using currently available tools.

In patients with suboptimal disease control and/or functional or biological signs of disease activity, the role of the small airways should be assessed to provide tailored treatment.

The choice of large-particle inhaled formulations over small-particle inhaled formulations must reflect the physician’s considerations of disease features, phenotype, and response to previous therapy.

## Abbreviations

ARIA: Allergic rhinitis and its impact on asthma
COPD: Chronic obstructive pulmonary disease
CT: Computed tomography
FEF 25-75 %: Forced expiratory flow at 25-75 % of forced vital capacity
FEV1: Forced expiratory volume in 1 second
GA2LEN: Global Allergy And Asthma European Network
ICS: Inhaled corticosteroid
MRI: Magnetic resonance imaging
WAO: World Allergy Organization
